# Diversity and structure of the rhizosphere microbial communities of wild and cultivated ginseng

**DOI:** 10.1186/s12866-021-02421-w

**Published:** 2022-01-03

**Authors:** Xiaoxue Fang, Huaying Wang, Ling Zhao, Manqi Wang, Mingzhou Sun

**Affiliations:** Key Laboratory of Molecular Epigenetics of Ministry of Education, Northeast Normal University, 130024 Changchun, China

**Keywords:** bacteria, fungi, wild ginseng, rhizosphere, soil, *Panax ginseng*

## Abstract

**Background:**

The resources of wild ginseng have been reducing sharply, and it is mainly dependent on artificial cultivation in China, Korea and Japan. Based on cultivation modes, cultivated ginseng include understory wild ginseng (the seeds or seedlings of cultivated ginseng were planted under the theropencedrymion without human intervention) and farmland cultivated ginseng (grown in farmland with human intervention). Cultivated ginseng, can only be planted on the same plot of land consecutively for several years owing to soilborne diseases, which is mainly because of the variation in the soil microbial community. In contrast, wild ginseng can grow for hundreds of years. However, the knowledge of rhizosphere microbe communities of the wild ginseng is limited.

**Result:**

In the present study, the microbial communities in rhizosphere soils of the three types of ginseng were analyzed by high-throughput sequencing of 16 S rRNA for bacteria and internal transcribed spacer (ITS) region for fungi. In total, 4,381 bacterial operational taxonomic units (OTUs) and 2,679 fungal OTUs were identified in rhizosphere soils of the three types of ginseng. Among them, the shared bacterial OTUs was more than fungal OTUs by the three types of ginseng, revealing fungal communities were to be more affected than bacterial communities. In addition, the composition of rhizosphere microbial communities and bacterial diversity were similar between understory wild ginseng and wild ginseng. However, higher bacterial diversity and lower fungal diversity were found in rhizosphere soils of wild ginseng compared with farmland cultivated ginseng. Furthermore, the relative abundance of Chloroflexi, *Fusarium* and *Alternaria* were higher in farmland cultivated ginseng compared to wild ginseng and understory wild ginseng.

**Conclusions:**

Our results showed that composition and diversity of rhizosphere microbial communities were significantly different in three types of ginseng. This study extended the knowledge pedigree of the microbial diversity populating rhizospheres, and provided insights into resolving the limiting bottleneck on the sustainable development of *P. ginseng* crops, and even the other crops of *Panax*.

**Supplementary Information:**

The online version contains supplementary material available at 10.1186/s12866-021-02421-w.

## Background

The rhizosphere is an active area of soil where plant roots and microorganisms interact, and is of great importance for plant health, development, productivity as well as for nutrient cycling [1, 2]. The microbiome can be beneficial or harmful to the host plants. Beneficial microorganisms provide nutrients from the soil and protect the plant from pathogens, enhancing tolerance to abiotic stresses [3]. However, pathogenic microorganisms slow down plant growth, reduce survival rate, and cause yield loss [4]. In recent years, many studies have been conducted to characterize rhizosphere microbiome in plant varieties, such as barley, wheat, soybean and blueberry [5–8]. These studies mainly showed that plant varieties can affect the rhizosphere microbial diversity and composition. Therefore, a better understanding the effects of the composition and function in the rhizosphere microbial community will contribute to plant breeding and open up a new approach for the rational use of plant-microbial interactions in agriculture.

The ginseng (*Panax ginseng* C. A. Meyer.) is used as a traditional Chinese medicine to treat many diseases, due to its anti-inflammatory and antitumor compounds [9]. Ginseng belongs to the family Araliaceae that is distributed in Asia, particularly in Korea and China [10]. Wild ginseng germplasm resources are scarce due to excessive land exploitation and disruption of the environment; thus, wild ginseng has been gradually replaced by cultivated ginseng in the market [11]. There are two kinds of cultivated ginseng, farmland cultivated ginseng and understory wild ginseng. Farmland cultivated ginseng is planted in farmland that was once forested with agricultural management, such as artificial shading and spraying pesticides, and farmland cultivated ginseng grows very quickly. In contrast, understory wild ginseng refers to cultivated ginseng seeds or seedlings grown under natural forest conditions for many years with little human interference. Its morphology and intrinsic quality characteristics of roots are similar to those of wild ginseng [12].

Cultivated ginseng, especially farmland cultivated ginseng, is susceptible to various soil-borne diseases. Farmland cultivated ginseng roots are harvested 5–6 years after planting, and the survival rate of farmland cultivated ginseng seedlings was less than 25% after 3 years [13]. Previous studies have showed Chloroflexi and *Fusarium* can cause root rot and root rust, and that these are the two most common soil-borne diseases, resulting in reduced ginseng quality and production [14–16]. In addition, root rot and root rust are also catastrophic diseases of other crops of *Panax*, like *Panax quinquefolium* and *P. notoginseng* [17, 18]. So bacterial and fungal community in rhizosphere soils changes during cultivation of these crops had received much attention [19]. It has been shown that microbial community in rhizosphere soil of ginseng is affected by cultivation ages, developmental stages and cultivation modes [13, 20–22]. Dong et al. (2017) found that fungal diversity increased, whereas bacterial diversity decreased in the rhizosphere soils at the root growth stage of ginseng [22]. Dong et al. (2018) also found that soil bacterial diversity decreased with the increase of ginseng planting years by comparing 1, 2 and 3 year old ginseng [21].

However, wild ginseng can grow in natural environments for decades or even hundreds of years [23]. Recent findings suggested that, one of the roles of rhizosphere microorganisms is to protect plants from pathogen infection by promoting the cascade modification of beneficial microorganism [24, 25]. These raise the question: the root microorganisms of wild ginseng are different from cultivated ginseng, and can this difference protect wild ginseng from pathogenic microbes? Whereas the structure in the rhizosphere microbial communities of wild ginseng is not clear. Therefore, in this study, we explored the diversity and structure of the bacterial and fungal communities in rhizosphere soil of wild ginseng, and also compared the rhizosphere microbial communities of wild ginseng and two types of cultivated ginseng. The study will provide insights into underlying mechanisms of ginseng planting and disease resistance.

## Results

### Rhizosphere community diversity in three types of ginseng

**Fig. 1:**
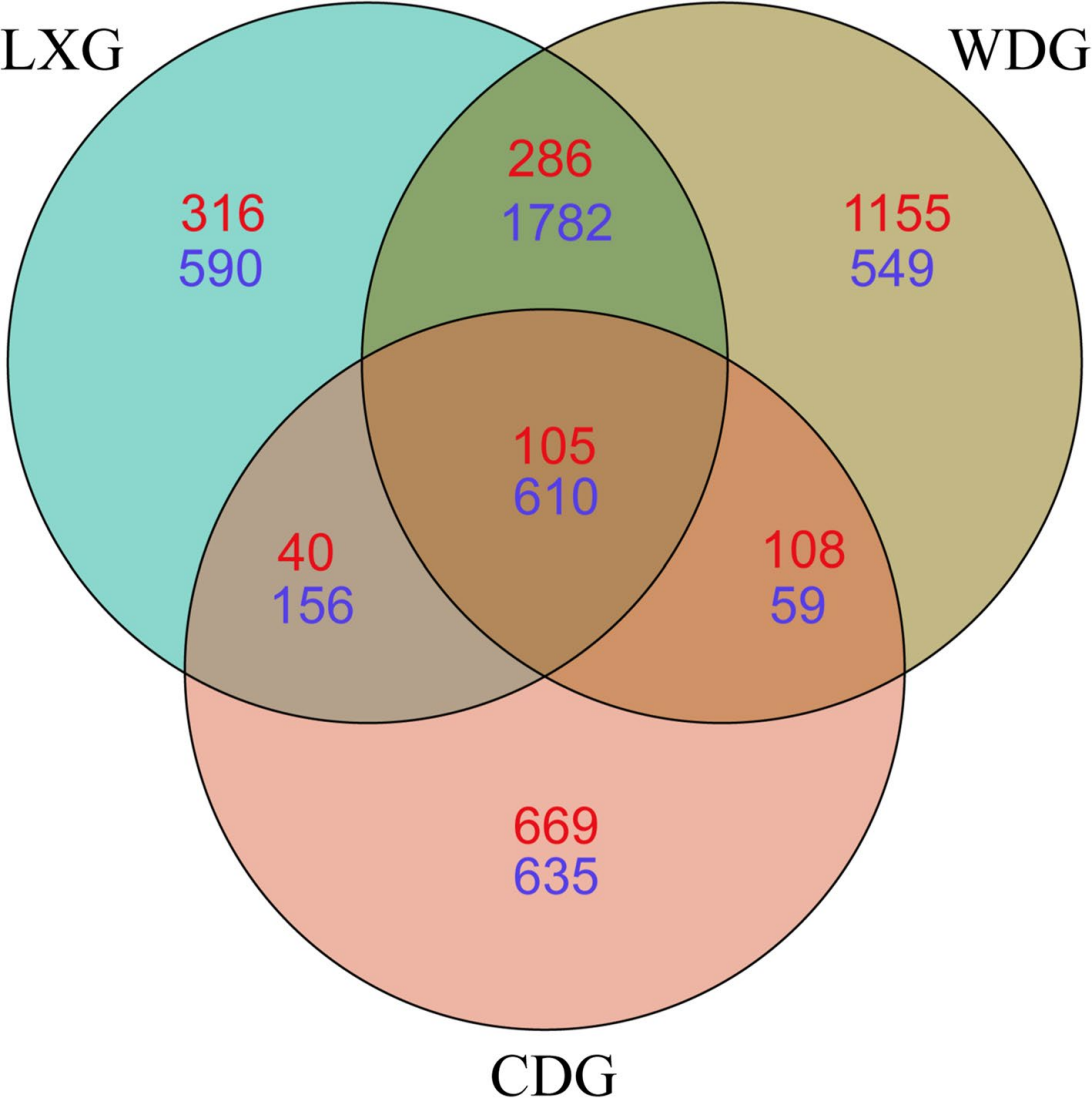
Venn diagrams of shared bacterial (blue) and fungal (red) OTUs in rhizosphere of three types of ginseng. LXG, understory wild ginseng; CDG, farmland cultivated ginseng; WDG, wild ginseng

To explore the bacterial and fungal communities in the rhizosphere soil of understory wild ginseng (LXG), farmland cultivated ginseng (CDG) and wild ginseng (WDG), we obtained 1,135,354 high-quality paired reads by high-throughput sequencing of 16S rRNA for bacteria and 797,696 paired reads by high-throughput sequencing of internal transcribed spacer (ITS) region for fungi. In total, 3,138 bacterial operational taxonomic units (OTUs) and 747 fungal OTUs were identified in LXG, 1,460 bacterial OTUs and 922 fungal OTUs were identified in CDG, while 3,000 bacterial and 1,654 fungal OTUs were identified in WDG, respectively (Fig. 1). The percentage of shared OTUs between LXG and WDG was smaller for the fungal community than for the bacterial community, and this pattern also occurred between LXG and CDG and between WDG and CDG (Fig. 1).

**Fig. 2:**
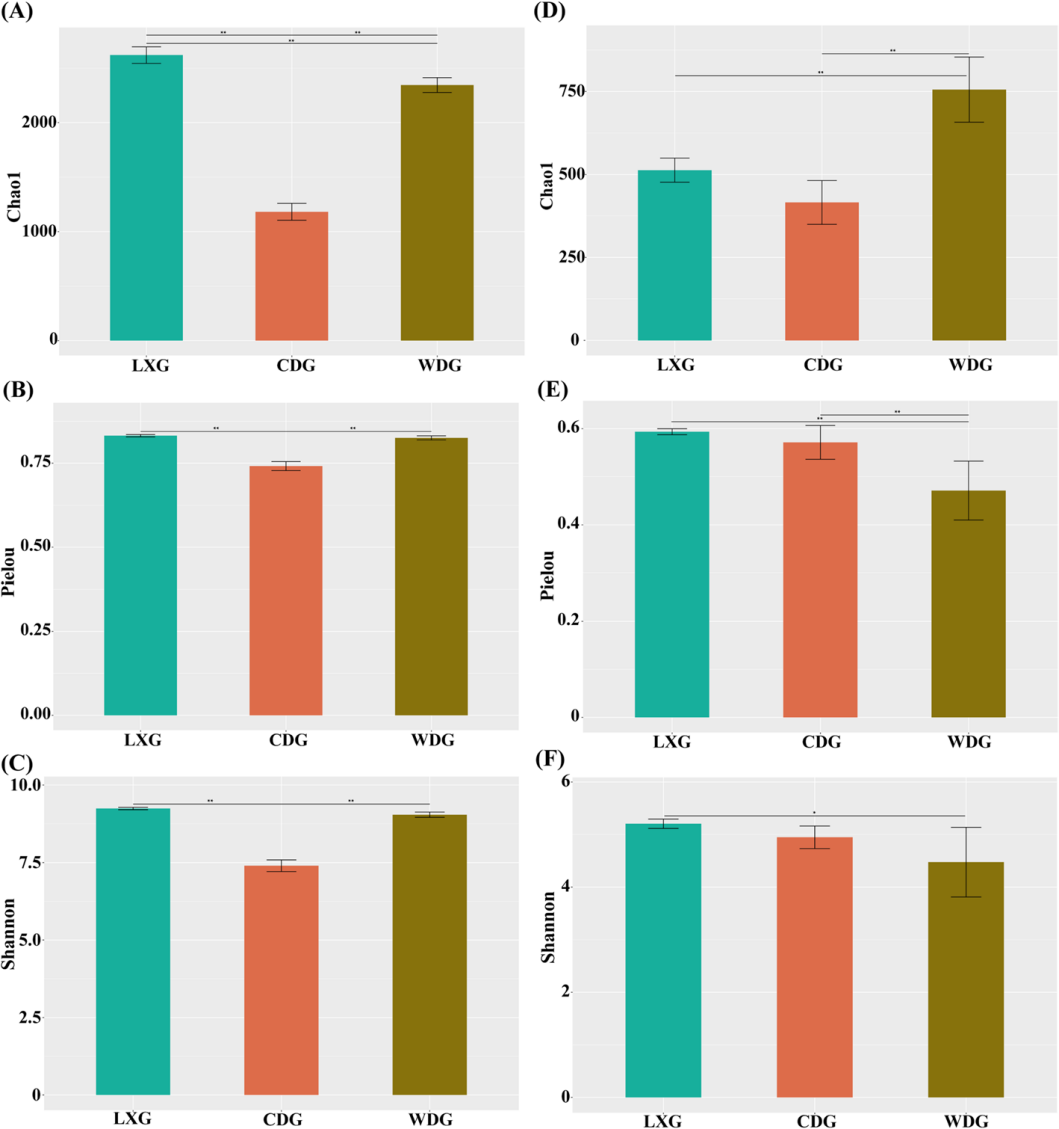
Chao 1, Pielou and Simpson indexes in the bacteria (**A**), (**B**), (**C**) and fungi (**D**), (**E**), (**F**) from three types of ginseng in rhizosphere, data were means ± standard error. LXG, understory wild ginseng; CDG, farmland cultivated ginseng; WDG, wild ginseng. *Significant at the 0.05 probability level. **Significant at the 0.01 probability level

The rhizosphere microbial diversity differed among three types of ginseng (Fig. 2). The bacterial alpha diversity of LXG and WDG were similar, however, that of CDG was significantly lower than those of WDG (*p* < 0.01) (Fig. 2 A, B, C). WDG had the highest fungal species richness (Chao 1), however, the fungal species evenness (Pielou) and fungal species diversity (Shannon) of WDG was the lowest (*p* < 0.01) (Fig. 2D, E, F). The fungal alpha diversity of LXG was higher than that of CDG (Fig. 2D, E, F).

### Bacterial and fungal community composition

**Fig. 3:**
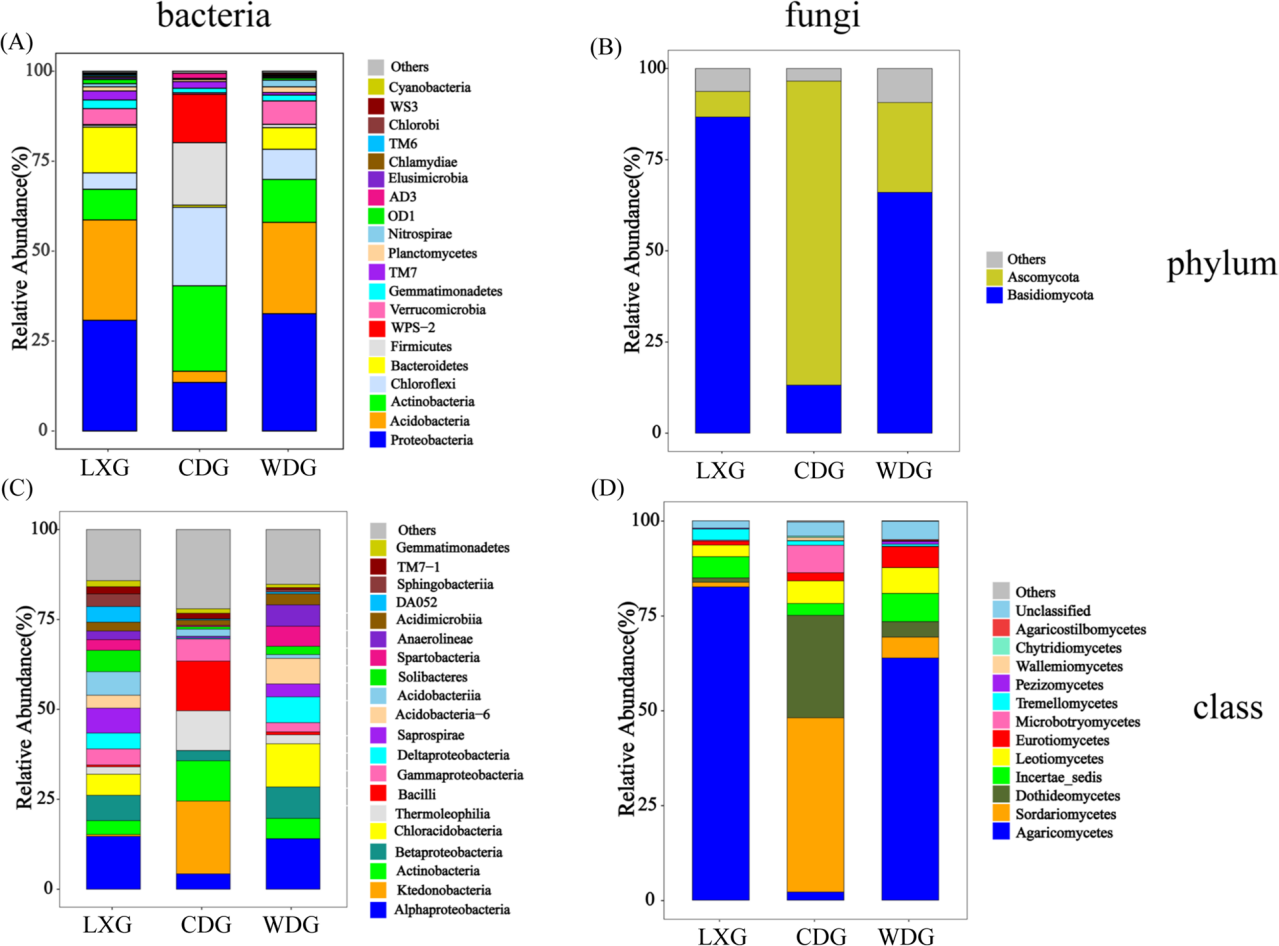
The composition of bacterial and fungal community from different types of ginseng rhizosphere. The phylum level of bacteria (**A**) and fungi (**B**), and the class level of bacteria (**C**) and fungi (**D**). The relative abundances in the top 20 were chosen to exhibit. Others represented of low relative abundance that ranks lower than top 20. LXG, understory wild ginseng; CDG, farmland cultivated ginseng; WDG, wild ginseng

The composition and abundance for each taxon were obtained based on the OTU classification results. For bacteria, the dominant phyla were Proteobacteria, Acidobacteria, Actinobacteria and Chloroflexi in LXG (relative abundances of 30.76%, 27.92%, 8.51% and 4.57%, respectively) and WDG (relative abundances of 32.61%, 25.39%, 11.92% and 8.39%, respectively) (Fig. 3 A), However, Actinobacteria (23.75%) was the most phylum in CDG, followed by Chloroflexi (21.85%), Firmicutes (17.40%) and Proteobacteria (13.53%). The ANOVA analysis suggested that the proportions of each main four phyla were not significantly different between LXG and WDG, however, these four phyla (Proteobacteria, Acidobacteria, Actinobacteria and Chloroflexi) were significantly different between WDG and CDG, LXG and CDG, respectively (Additional files 2: Fig. S2, *p* < 0.01). The most abundant bacterial classes were Alphaproteobacteria and Betaproteobacteria in LXG (relative abundances of 14.71% and 7.07%, respectively) and WDG (relative abundances of 14.02% and 8.76%, respectively), then Saprospirae (6.94%) and Acidobacteria (6.94%) in LXG, after Chloracidobacteria (12.02%) and Deltaproteobacteria (7.17%) in WDG. Ktedonobacteria (20.24%), Bacilli (13.80%), Actinobacteria (11.18%) and Thermoleophilia (11.05%) had the highest relative abundances in CDG (Fig. 3 C).

**Fig. 4:**
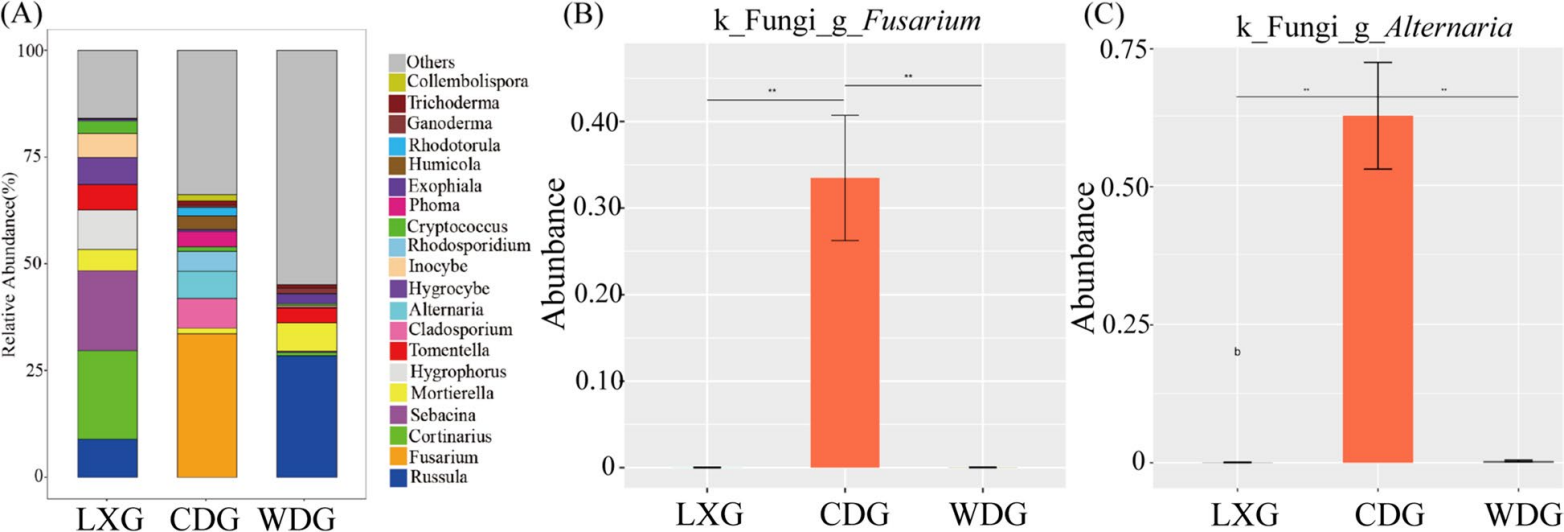
The composition of fungal community at the genus level (**A**). The relative abundances in the top 20 were chosen to exhibit. Others represented of low relative abundance that ranks lower than top 20. The relative abundance of *Fusarium* (**B**) and *Alternaria* (**C**) in the rhizosphere for three types of ginseng, data were means ± standard error. LXG, understory wild ginseng; CDG, farmland cultivated ginseng; WDG, wild ginseng. *Significant at the 0.05 probability level. **Significant at the 0.01 probability level

For fungi, Ascomycota and Basidiomycota accounted for more than 90% of the total abundance across LXG, CDG and WDG (Fig. 3B), but the result of ANOVA indicated that the proportions of each phylum were different in each type of ginseng (Additional files 2: Fig. S3, *p* < 0.01). At the class level, the dominant classes were Agaricomycetes, incertae_sedis and Leotiomycetes in LXG (relative abundances of 82.56%, 5.61% and 3.04%, respectively) and WDG (relative abundances of 63.85%, 7.45% and 6.78%, respectively) (Fig. 3D). In CDG, the relative abundance of Sordariomycetes (45.92%) was the highest, followed by Dothideomycetes (27.00%) and Microbotryomycetes (7.22%) (Fig. 3D). At the genus level, the most abundant genus was *Fusarium* (28.37%) in CDG; however, it was rare in LXG (< 0.01%) and WDG (0.01%) (Fig. 4 A), and ANOVA suggested that the abundance of *Fusarium* was the highest in CDG (*p* < 0.01) (Fig. 4B). In addition, the abundance of *Alternaria* in CDG was higher than in LXG and WDG (*p* < 0.01) (Fig. 4 C).

**Fig. 5:**
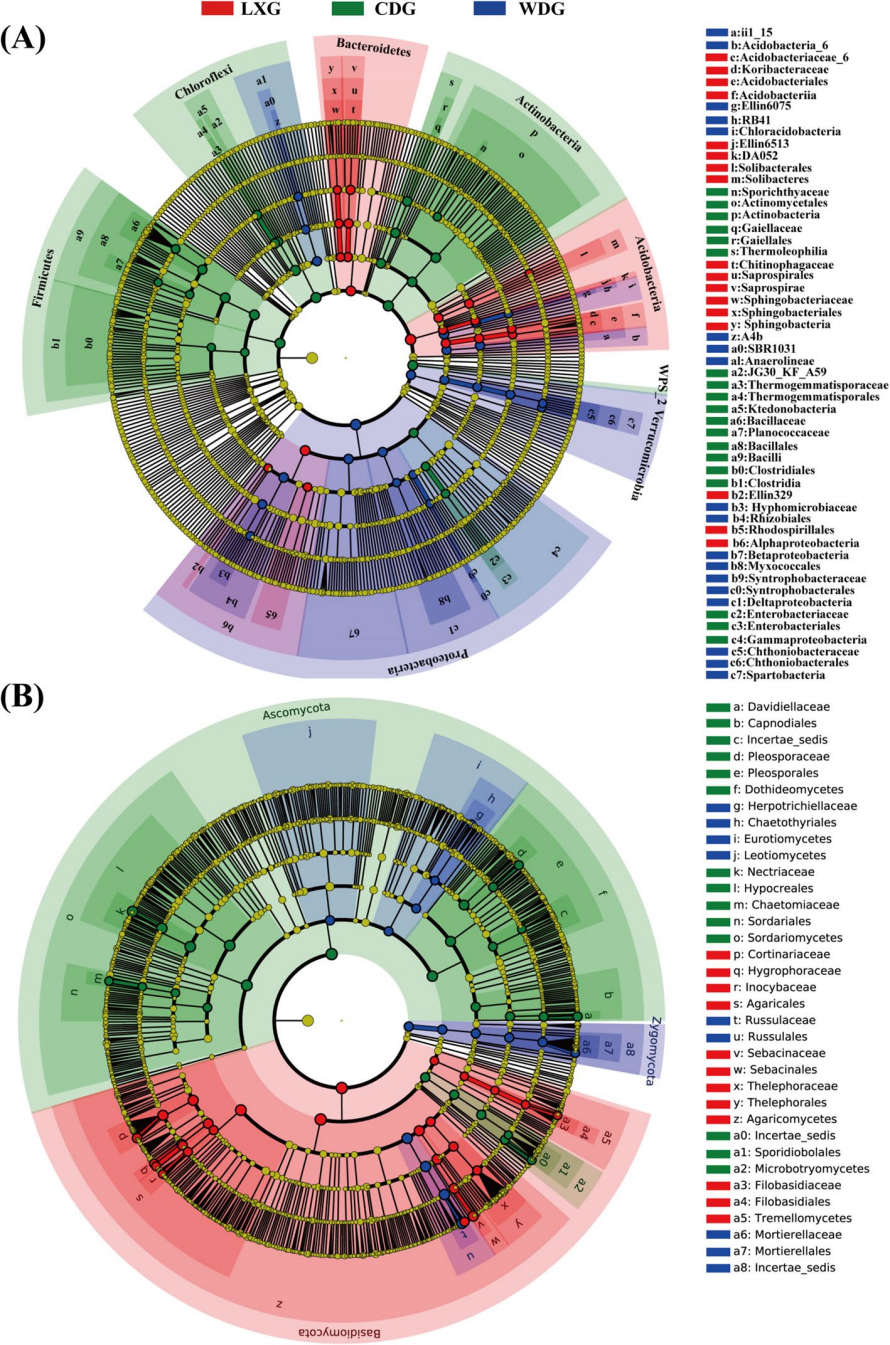
LEfSe analysis showing the different rhizosphere taxa among three types of ginseng in bacteria (**A**) and fungi (**B**). The diameter of each circle is proportional to the relative abundance of the taxon. The inner to outer circle corresponds to the level of the phylum to the genus. LXG, understory wild ginseng; CDG, farmland cultivated ginseng; WDG, wild ginseng

The LEfSe analysis of the rhizosphere bacterial communities showed that there were 68 differentially abundant taxa among the three types of ginseng. Of the 68 taxa, 23 were differentially abundant in WDG (Fig. 5 A, Additional files 2: Fig. S4A), namely the Verrucomicrobia phylum and the Deltaproteobacteria, Betaproteobacteria, Acidobacteris_6, Chloracidobacteria and Anaerolineae classes. The enriched taxa in LXG were the phyla Bacteroidetes and Acidobacteria and the class Alphaproteobacteria. The differentially abundant taxa in the rhizosphere soils of CDG were the Firmicutes, Actinobacteria, WPS-2 and Chloroflexi phyla and the Gammaproteobacteria class.

The LEfSe analysis of the fungal communities from LXG, CDG and WDG showed that, there were 69 differentially abundant taxa with an LDA score higher than 2.0 (Fig. 5B, Additional files 2: Fig. S4B). Among the 69 fungal taxa, 15 fungal taxa were differentially abundant in WDG, principally including the Mortierellomycota phylum, the Leotiomycetes and Eurotiomycetes classes, and the Russulales order. The abundant taxa in the rhizosphere soils of LXG were the Agaricomycetes and Tremellomycetes classes. The most differentially abundant fungal taxa were the Sordariomycetes, Dothideomycetes and Microbotryomycetes classes in CDG.

### Factors driving rhizosphere microbial communities in three types of ginsengs

Effects of soil physical and chemical properties and plant types on the structure of microbial communities in the rhizosphere of ginseng were analyzed using 4, 381 bacterial OTUs and 2, 679 fungal OTUs. The result of mantel test showed that bacterial and fungal community were correlated with soil physical and chemical properties, but not significant (Table 1, *p* > 0.05). However, the PERMANOVAs results suggested ginseng type explained 90.118% and 84.699% of variance in bacterial and fungal communities, respectively (Table 2, *p* < 0.01). The PCoA of Bray-Curtis distance matrix demonstrated that samples of LXG, CDG and WDG showed clear separation, suggesting that the bacterial and fungal communities were obviously different among three types of ginseng (Additional files 2: Fig. S1).

**Table 1:** Mantel tests of the influence of soil physical and chemical properties on microbial communities associated

	bacteria		fungi	
Soil parameter	R^2^	p	R^2^	p
TP	-0.45	0.67	-0.93	1.00
TN	-0.45	0.67	-0.93	1.00
TK	-0.45	0.67	-0.93	1.00
pH	1.00	0.34	0.78	0.33
Sand	0.89	0.17	0.36	0.50
Silt	0.89	0.17	0.35	0.50
Clay	-0.99	1.00	-0.63	0.83

Note: TP, total phosphorus; TN, total nitrogen; TK, total potassium; Sand, the content of sand in soil; Silt, the content of silt in soil; Clay, the content of clay in soil; R^2^, correlation coefficient; p, p-value

**Table 2:** PERMANOVAs of the influence of ginseng types on microbial communities associated

	bacterial community			fungal community		
factor	F	R^2^	p	F	R^2^	p
types	68.3980	0.90118	< 0.01	41.5160	0.84699	< 0.01

Note: F, F.model; R^2^, Variation ; p, p-value

## Discussion

### The factor affected the rhizosphere microbial community

Owing to the lack of wild ginseng germplasm resources and the low survival rate of cultivated ginseng, it is very difficult to collect the rhizosphere soils of cultivated ginseng and wild ginseng in the same field. This phenomenon is common among wild species. Wang et al. (2018) compared the rhizosphere bacterial diversity of four *Ferulic* species from four locations based on 16S rRNA sequencing [26]. Nevertheless, the three sampling locations of our study were all in Jilin Province of China. In addition, we also downloaded the data of soil physical and chemical properties to explore the influence of soil physical and chemical properties on microbial communities. The mantel test suggested soil physical and chemical properties effected bacterial and fungal communities, but not significant. However, PERMANOVAs result indicated that the ginseng types significantly affected the rhizosphere microbial community. Christine et al. (2007) found that there was no difference in soil microbial characteristics between organic soil and conventional fertilized soil, and the type of plant has a great influence on microbial biomass [27]. Muhammad et al. (2020) also suggested plant types had stronger effects on soil microbial communities than biochar or fertilizer [28]. Therefore, we considered that the effect of soil physical and chemical properties on bacterial and fungal communities is not obvious. The type of ginseng might have a great influence on the rhizosphere microbial community.

In our study, we found that the proportion of shared fungal OTUs was much smaller than that of bacterial OTUs between any two pairs of LXG, CDG and WDG. We inferred fungal communities appeared to be more affected than bacterial communities. Coleman-Derr et al. (2016) suggested that fungal communities were perhaps more shaped by geographic distance than bacterial communities in rhizosphere soils of *Agave* from California and Mexico [29]. Wang et al. (2021) also found the fungal communities were more influenced than the bacterial communities by different ginseng cultivars [30]. These studies showed that fungal communities are more affected than bacterial communities, which is consistent with our results.

### Different bacterial diversity and fungal diversity in three types of ginsengs

The bacterial alpha diversity of LXG and WDG were similar, and WDG shared more bacterial OTUs with LXG than with CDG. Maybe the understory wild ginseng and wild ginseng both grow in the theropencedrymion, moreover, understory wild ginseng is a semi-wild ginseng and its morphology and intrinsic quality characteristics of roots are similar to those of wild ginseng [12, 31]. However, the bacterial alpha diversity of CDG decreased compared with that of WDG, showing a loss of natural bacterial diversity in the rhizosphere. A number of agronomic management practices potentially influenced bacterial diversity in farmland cultivated ginseng. Ginseng is a shade-loving plant, so farmland cultivated ginseng need to be artificially shaded and irrigated during the growth process [32]. These treatments effected the soil moisture and regional temperature of cultivated ginseng. Nuccio et al. (2016) compared rhizosphere microorganisms from three California prairie wild oats (*Avena* spp.), suggesting rhizosphere microorganisms were influenced by factors related to the regional climate (soil moisture and temperature) [33]. Therefore, artificial shading and irrigation treatment affected the bacterial diversity. In addition, farmland cultivated ginseng was susceptible to diseases during cultivation, so pesticides were sprayed to reduce the impact of pests and diseases [32, 34]. Pesticides might have indirectly affected root exudates or directly inhibited the reproduction of certain rhizosphere microorganisms during ginseng cultivation [35, 36]. Thus, pesticide disposal also reduced the microbial diversity of farmland cultivated ginseng. Furthermore, the decrease in bacterial diversity might also be associated to the host plants. The rhizosphere microbial diversity of domesticated sugar beets decreased compared to their wild ancestors [37]. Cultivated crops had the characteristics of fast growth and high yield, which might lead to different amounts and types of organic compounds secreted from the roots, resulting in different subsurface microbial community structures [38].

In contrast, fungal diversity showed the opposite phenomenon. We found that the fungal diversity (Shannon and Pielou) in CDG was higher than that in WDG. But the Chao 1 was the highest in WDG, which may be associated with the greater number of fungal OTUs in WDG than in CDG and LXG. A similar phenomenon has been observed in soybean and their wild species, and the fungal diversity of cultivated soybean increased compared to its wild type [39]. Selective breeding of modern crops perhaps had promoted the proliferation of specific crop-related microbial taxa, leading to increased fungal diversity in cultivated ginseng [40]. Furthermore, the fungal alpha diversity of LXG was higher than CDG, which might be related to high content of ginsenoside for understory wild ginseng. According to previous studies, the fungal diversity of soil was affected by the content of ginsenosides in ginseng growth [41, 42]. Yong et al. (2007) also found understory wild ginseng contained higher amounts of ginsenosides than farmland cultivated ginseng [43]. Therefore, understory wild ginseng secreted higher ginsenosides to the rhizosphere soil, resulting in higher diversity of rhizosphere fungi.

### Changes in the composition of microbial communities among the three types of ginsengs

In the composition of bacterial communities, our study indicated that Proteobacteria and Acidobacteria all existed in LXG, CDG and WDG, and previous studies also confirmed that Proteobacteria and Acidobacteria were the dominant populations in the rhizosphere soil of ginseng [44]. The relative abundance of Chloroflexi in CDG was higher than that in WDG and LXG (*p* < 0.01). Wang et al. (2019) suggested that root rust may be caused by Chloroflexi in rhizosphere microbial communities based on five cultivated ginseng samples with different severity of rusty root disease [15]. Although the rhizosphere soils in our study came from healthy ginseng, Chloroflexi was a hidden danger that causes the disease of farmland cultivated ginseng. In addition, Verrucomicrobia was significantly high abundance in WDG and also has been found in the rhizosphere of the common bean, and Verrucomicrobia was also mainly found in wild bean accessions [38]. Verrucomicrobia probably had established beneficial links with wild species to protect wild species from pathogens. However, there are few studies on Verrucomicrobia, which still need further research.

In fungi community, the same fungal phyla were detected but the fungal community composition, also at phylum level, the relatively abundances of Basidiomycota and Ascomycota was different in LXG, CDG and WDG. Ascomycota and Basidiomycota also were the dominant phyla in the rhizosphere soil of *P. notoginseng* [45]. Ascomycota, which has an important role in the decomposition of soil organic matter and largely dominates the active fungal community through the assimilation of root exudates [46]. Furthermore, the main pathogenic fungus *Fusarium* that caused root rot belongs to the Ascomycota, which was the most predominant phylum in CDG. *Fusarium* was a potential phytopathogen (includes potential pathogens) that can cause root rot in various species, including ginseng, American ginseng, soybean and sunflower [47–50]. We found that the abundance of *Fusarium* was the highest in CDG, while that in WDG was only 0.01% (*p* < 0.01). Likewise, cultivated rice had a higher abundance of pathogens comparing with the wild varieties [39]. Moreover, *Alternaria* was a pathogenic fungi associated with ginseng rusty roots [51]. And the abundance of *Alternaria* in CDG was higher than that of LXG and WDG. This further suggests that domesticated crops reduced their ability to establish beneficial associations with the rhizosphere microbiome, thus stimulating the spread of pathogens in rhizosphere [38]. This also explains how wild ginseng can grow in natural environments for decades or even hundreds of years. In addition, continuous planting may also lead to an increase in rhizosphere pathogens. The pathogens, including *Alternaria* and *Fusarium*, that were highly enriched in the rhizosphere of 30-year sugar beet [52]. This result provided new scientific insights for the healthy growth, reducing the incidence of ginseng and improving the yield of ginseng. Through a comprehensive understanding of wild ginseng rhizosphere microorganisms, farmland soil can be improved to create a suitable soil environment for ginseng growth and reduce forest logging during ginseng planting.

## Conclusions

In this study, we systematically studied the rhizosphere microbial communities of three types of ginseng, including LXG, CDG and WDG. The results showed soil physical and chemical properties effected bacterial and fungal communities, but not significantly. However, the type of ginseng had a great influence on rhizosphere microbial communities. We found fungal communities were more susceptible than bacterial communities. By comparing rhizosphere microbe of three types of ginseng, the composition of rhizosphere microbial communities in LXG and in WDG was similar. There were significant differences in the composition of rhizosphere microbial community in WDG and in CDG. In addition, the bacterial diversity of WDG and LXG was also similar. The higher bacterial diversity and lower fungal diversity in WDG compared with CDG. Furthermore, the relative abundance of Chloroflexi, *Fusarium* and *Alternaria* were higher in CDG compared to WDG and LXG. This result may provide insights for ginseng breeding and yield improvement, and supply feasible information for soil management.

## Methods

### Sampling Sites and Samples Collection

Rhizosphere soil samples were collected from three type of ginsengs, including understory wild ginseng (LXG), farmland cultivated ginseng (CDG) and wild ginseng (WDG). Rhizosphere soil samples in this study were collected in August 2018. A collection of ginseng rhizosphere soil was made, and samples were taken from a depth of 20 cm using a sterile shovel. Ginseng plants were carefully removed from the ground, keeping the root system intact. The large clumps of soil on the roots were removed, then brushed soil attached to the roots with a brush. Each soil samples were passed through a 2 mm sieve, finally into a sterile tube. The soil samples in each type are from at least three healthy, disease-free roots of ginseng (one to three rhizosphere soil samples were collected from the roots of each ginseng). In total, the rhizosphere soils samples of LXG, CDG and WDG were set up with seven, five and six, respectively (Additional files 1: Table S1). All samples were then transported to the liquid nitrogen within one hour and immediately transported to the laboratory. Finally, the soil samples were stored at −80 °C until genomic DNA extraction using an E.Z.N.A.® Stool DNA Kit (Omega, Shanghai).

All ginsengs were grown for about 15 years. wild ginseng and understory wild ginseng both grow in theropencedrymion, and there were similar vegetation types under the forest. The rhizosphere samples of understory wild ginseng were collected from Linjiang city of Jilin Province, and the soil was Mollic Albi-boric Cambosols (sand 51%, silt 32%, clay 17%), then chemical characteristics of the soil were (mg kg^−1^): 82 (P), 199 (N) and 2198 (K) with pH (5.99). The soil samples of farmland cultivated ginseng were from Ji’an city of Jilin Province, and the soil was Mollic bori-Udic Cambosols (sand 33%, silt 49%, clay 18%), and chemical characteristics of the soil were (mg kg^−1^): 146 (P), 401 (N) and 1823 (K) with pH (6.62). The rhizosphere soils of wild ginseng were collected from Korean autonomous county, Jilin Province. The soil was Mollic bori-Udic Cambosols (sand 45%, silt 37%, clay 18%), and chemical characteristics of the soil were (mg kg^−1^): 146 (P), 401(N) and 1823 (K) with pH (5.99) (Table 3). The soil characteries were mapped using the National Earth System Science Data Center [53].

**Table 3:** The physical and chemical properties of the soil in sampling locations for ginseng

	LXG	CDG	WDG
soil taxonomy	Mollic Albi-boric Argosolos	Mollic bori-Udic Cambosols	Mollic bori-Udic Cambosols
TP (mg kg^−1^)	82	146	146
TN (mg kg^−1^)	199	401	401
TK (mg kg^−1^)	2198	1823	1823
pH	5.99	6.62	5.99
Sand (%)	51	33	45
Silt (%)	32	49	37
Clay (%)	17	18	18

Note: TP, total phosphorus; TN, total nitrogen; TK, total potassium; Sand, the content of sand in soil; Silt, the content of silt in soil; Clay, the content of clay in soil. LXG, understory wild ginseng; CDG, farmland cultivated ginseng; WDG, wild ginseng

### PCR, amplicon quantification, HiSeq library construction and sequencing

The variable V3-V4 region of the bacterial 16S rRNA gene and fungal ITS1 region were amplified from each sample with the primers pairs 341F (5’-ACTCCTACGGGAGGCAGCAG-3’) / 806R (5’-GGACTACHVGGGTWTCTAAT-3’) and ITS-1 (5’-CTTGGTCATTTAGAGGAAGTAA-3’)/ITS-2 (5’-GCTGCGTTCTTCATCGATGC-3’) [54, 55]. All PCRs reactions were performed using NEB Phusion High-Fidelity PCR Master Mix following the manufacturer’s recommendations. The PCR reaction system contained 30 ng of DNA, 4µL of PCR primer mix and 25µL of PCR Master Mix. The following PCR conditions were used: 98 °C for 3 min; followed by 30 cycles of 98 °C for 45 s, 55 °C for 45 s and 72 °C for 45 s; and a final extension of 72 °C for 7 min. Then, the PCR products integrity was subjected to 1% agarose gel electrophoresis and purified using Ampure XP beads (Beckman, America) to remove the unspecific products. The final library was quantitated in two ways: determination of the average molecule length using an Agilent 2100 bioanalyzer instrument (Agilent DNA 1000 reagents, America), and quantification of the library by real-time quantitative PCR. The qualified libraries were sequenced pair-end on the system with the sequencing strategy PE250 under the HiSeq platform (Illumina, America).

### Data analysis and statistics

After removing reads with ambiguous bases, an average Phred score less than 20 and the length lower than 10 bp, the remaining reads were merged into Tags based on overlapping regions using FLASH (fast length adjustment of short reads, v1.2.11) within paired-end reads [56]. Then, operational taxonomic units (OTUs) were clustered with a 97% similarity cut off by using UPARSE (v7.0.1090) [57]. The chimeric sequences were identified and removed using UCHIME software (v4.2.40) [58]. OTUs undoubtedly belonging to chloroplasts or mitochondria were also removed. Subsequently, the taxonomic classification of the representative sequence for each bacterial and fungal OTUs was annotated using Greengenes (v201304) and UNITE (v7.2) reference databases, respectively, with the RDP Classifier (Ribosomal Database Project, v2.2). The taxa with significant different in rhizosphere microbial community among three type of ginsengs was performed by ANOVA (One-way Analysis of Variance, *p* < 0.05) in SPSS_Statistics_23. A venn plotter was used to obtain the number of unique and common OTUs using the ‘VennDiagram’ R package (v3.1.1). The alpha diversity of the bacterial and fungal communities was calculated with the Chao 1 (species richness), Pielou (species evenness) and Shannon (species diversity) indexes for each sample using in MOTHUR (v1.31.2) [59]. The differences in alpha diversity among the three types of ginseng were determined by Tukey’s honestly significant difference test in R package (v3.1.1) (*p* < 0.05). Mantel tests and Permutational ANOVAS (PERMANOVAs) were performed to assess the correlation between rhizosphere microbial communities and soil physical and chemical properties, type of ginseng by using R package ‘vegan’ (v2.5-7), respectively [60]. Principal coordinate analysis (PCoA) was performed in QIIME software (v1.80) to reflect the beta diversity of the microbial community, evaluate the similarity in community composition among the different types of ginseng based on the Bray-Curtis distance matrix [61]. Linear discriminant analysis (LDA) effect sizes (LEfSe) was used to detect notably different taxa among the samples using the Galaxy online analytics platform, and LEfSe identity different abundant taxa with an linear discriminant analysis (LDA) score higher than 2.0 (http://huttenhower.sph.havard.edu/galaxy).

## Supplementary Information

**Additional file 1.**

**Additional file 2.**

## Data Availability

The sequencing dataset analyzed during the current study is available in the NCBI Sequence Read Archive (PRJNA701796 and PRJNA701800).
