# Supplementary material for: Diversity and structure of the rhizosphere microbial communities of wild and cultivated ginseng
Source: BMC Microbiol. 2022 Jan 3;22:2. doi: 10.1186/s12866-021-02421-w (PMC8721995; doi:10.1186/s12866-021-02421-w)
Supplement: Supplementary file 1 — Additional file 1. [file 12866_2021_2421_MOESM1_ESM.docx]

**Supplementary table:**

|  | location | latitude | longitude | Ginseng types | n |
| --- | --- | --- | --- | --- | --- |
| LXG | Linjiang country | N41°75´36.86´´ | E127°60´29.35´´ | understory wild ginseng | 7 |
| CDG | Ji'an city | N41°31´54.46´´ | E125°91´48.33´´ | cultivate ginseng | 5 |
| WDG | Korean Autonomous County of Changbai | N41°31´19.31´´ | E127°57´07.38´´ | wild ginseng | 6 |

**Table S1.** Geographic location, types of ginsengs and the number of rhizosphere soil samples of three types of ginsengs.

Note: n, the number of rhizosphere soil samples; LXG, understory wild ginseng; CDG, farmland cultivated ginseng; WDG, wild ginseng.
