# Supplementary material for: Diversity and structure of the rhizosphere microbial communities of wild and cultivated ginseng
Source: BMC Microbiol. 2022 Jan 3;22:2. doi: 10.1186/s12866-021-02421-w (PMC8721995; doi:10.1186/s12866-021-02421-w)
Supplement: Supplementary file 2 — Additional file 2. [file 12866_2021_2421_MOESM2_ESM.docx]

**Supplementary figure:**


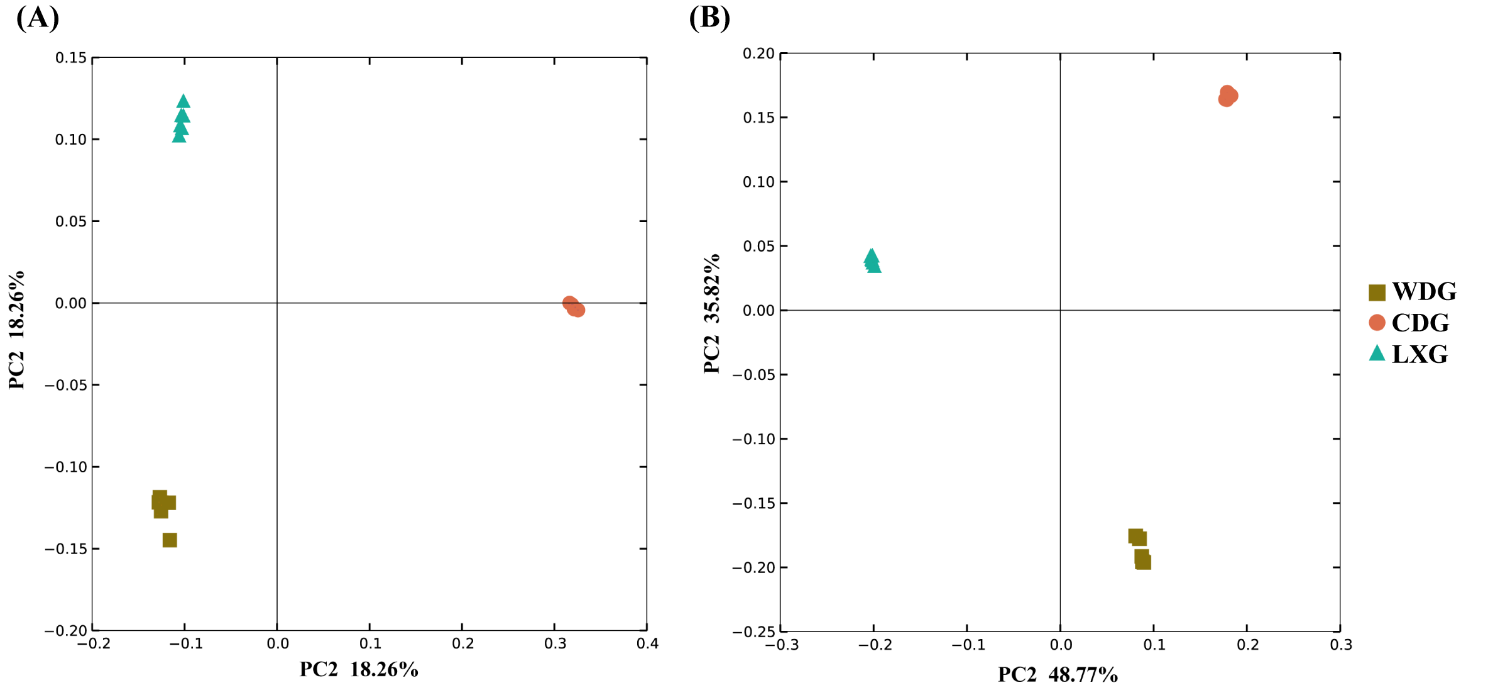


**Figure S1.** Principal component analysis (PCoA) based on the distance matrix calculated using the Bray-Curtis distance matrix for rhizosphere soil samples collected from the three types of ginseng. (A) and (B) represent the bacterial and fungal communities, respectively. LXG, understory wild ginseng; CDG, farmland cultivated ginseng; WDG, wild ginseng.


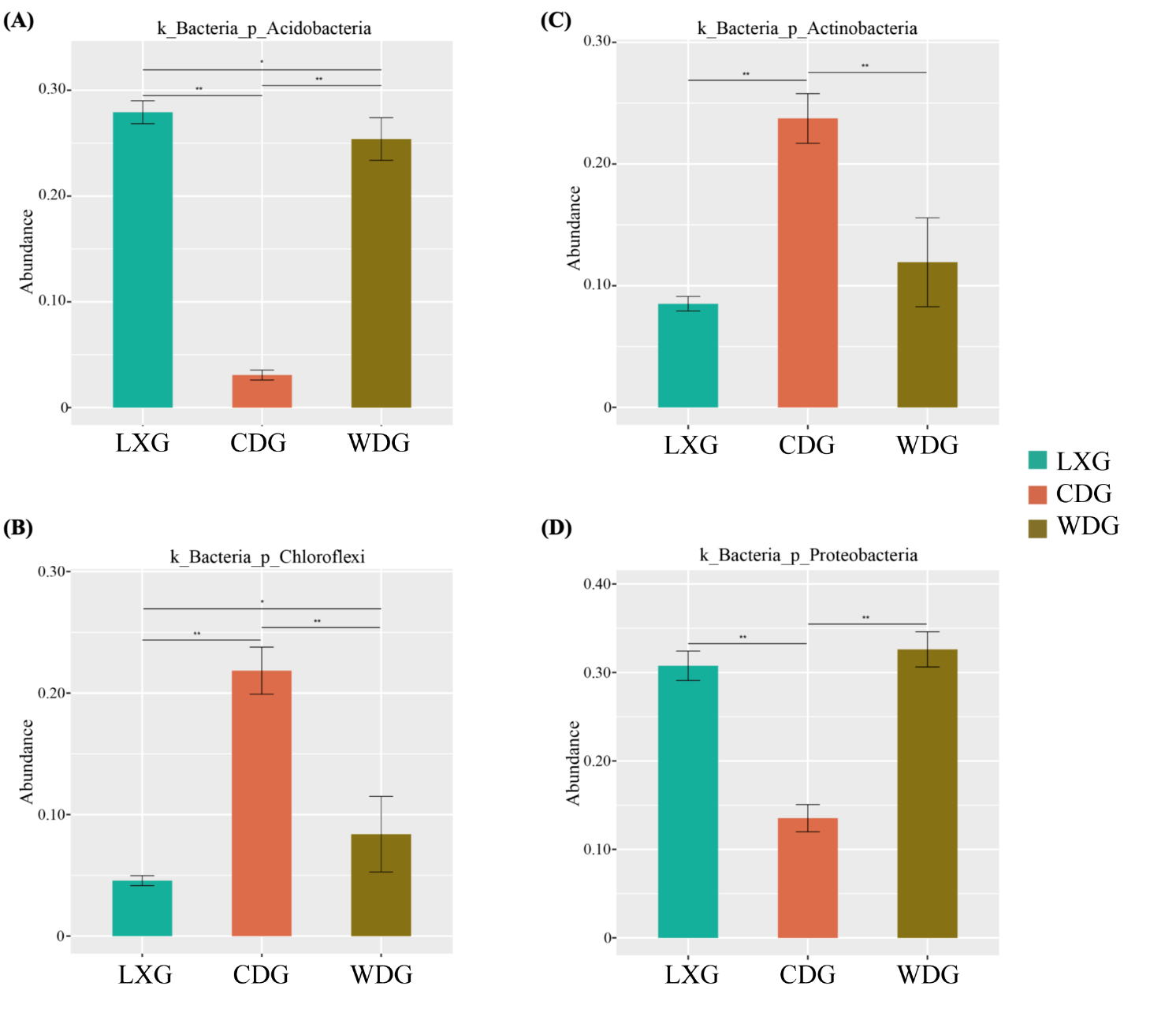


**Figure S2.** Relative abundance of the bacterial phyla in the rhizosphere from three types of ginseng, data were means ± standard error. (A), Acidobacteria; (B), Actinobacteria; (C), Chloroflexi; (D), Proteobacteria. LXG, understory wild ginseng; CDG, farmland cultivated ginseng; WDG, wild ginseng.

*Significant at the 0.05 probability level.

**Significant at the 0.01 probability level.


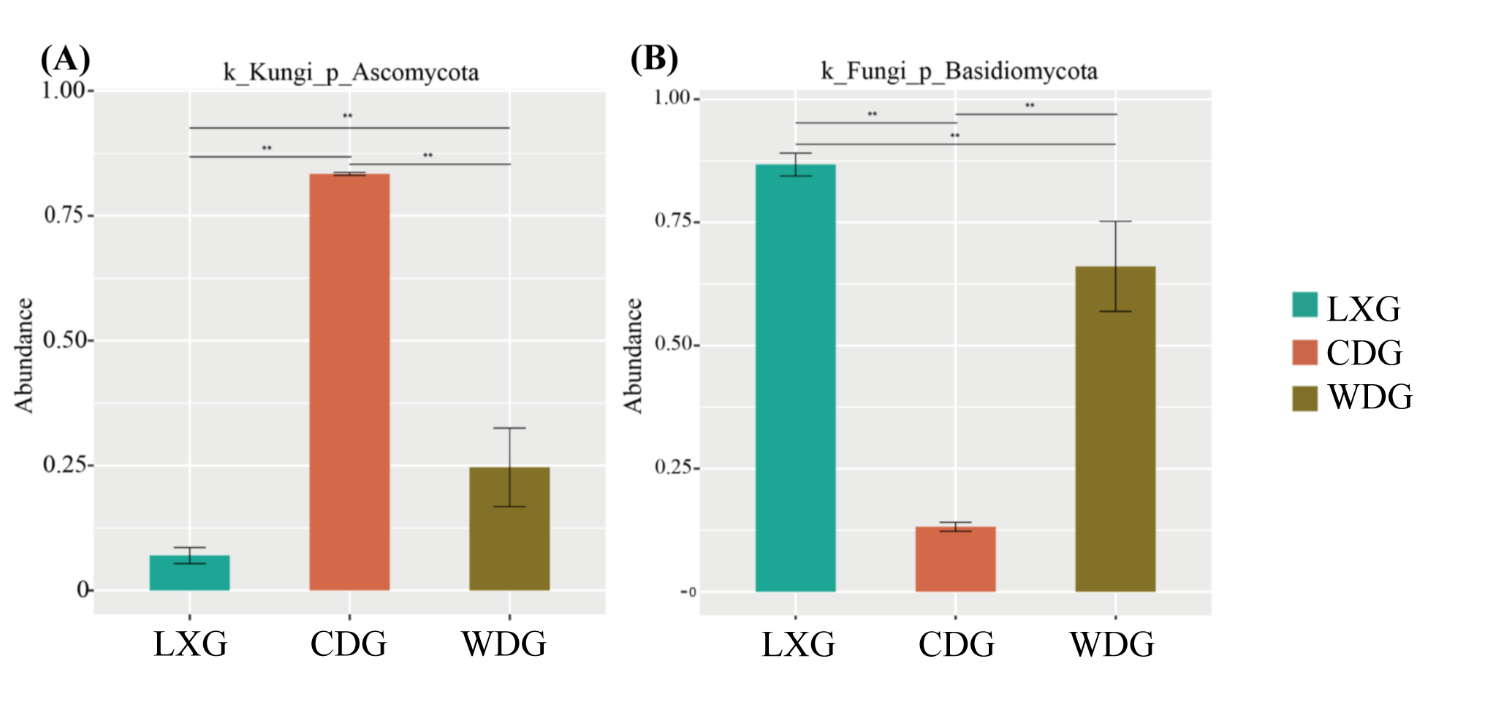
 **Figure S3.** Relative abundance of the fungal phyla in rhizosphere from three types of ginseng, data were means ± standard error. (A), Ascomycota; (B), Basidiomycota. LXG, understory wild ginseng; CDG, farmland cultivated ginseng; WDG, wild ginseng.

*Significant at the 0.05 probability level.

**Significant at the 0.01 probability level.


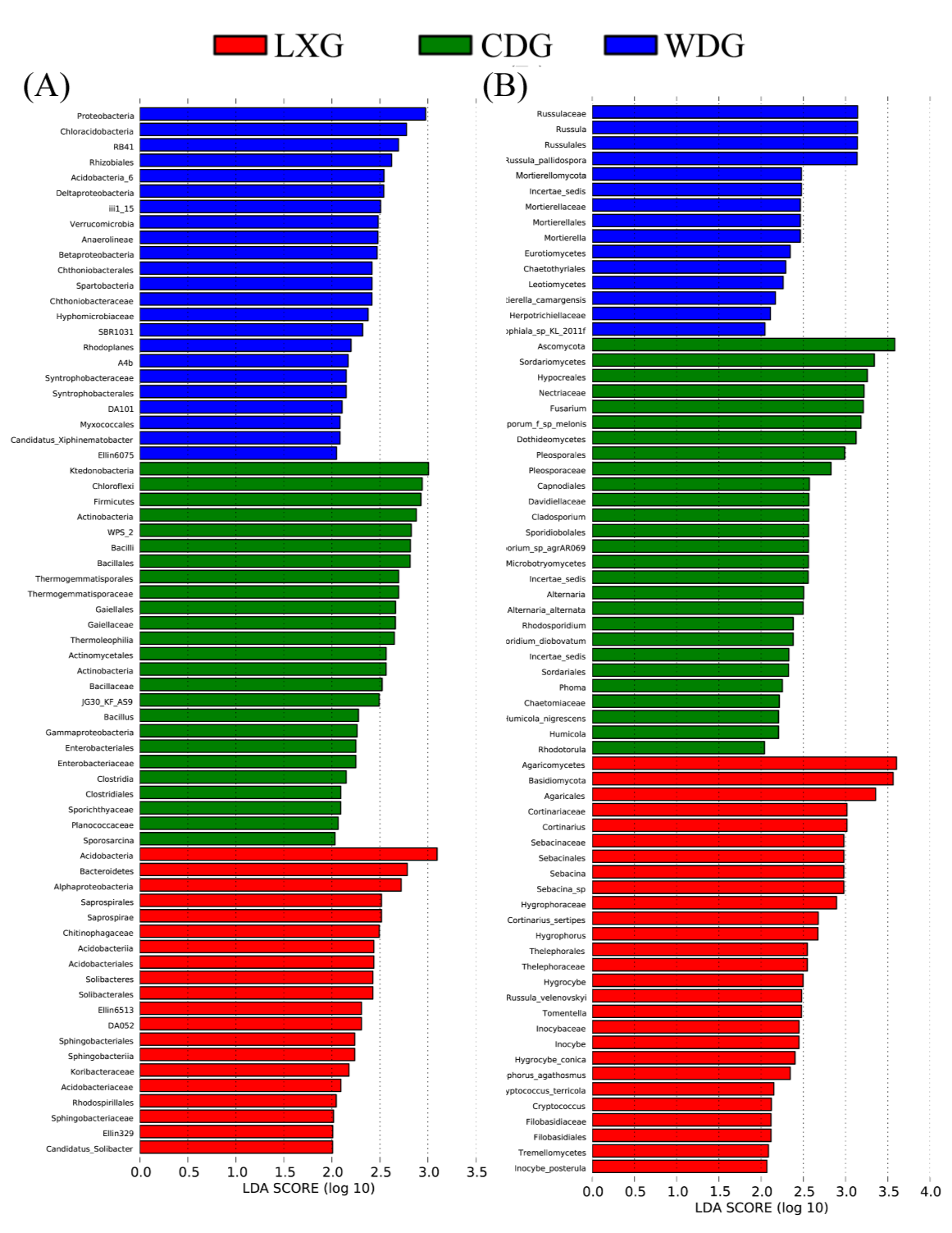


**Figure S4.** Linear discriminant analysis (LDA) scores of significant different taxa in the rhizospheres of three types of ginseng. The specific classification taxa name and LDA (LDA > 2.0) score were displayed in the panel. (A), bacteria; (B). fungi. Blue, green and red represent different biomarkers in WDG, CDG and LXG, respectively. LXG, understory wild ginseng; CDG, farmland cultivated ginseng; WDG, wild ginseng.
